# The Physical History of Various Nations of the Earth, with Special Reference to Their Teeth

**Published:** 1867-12

**Authors:** J. Allen


### ARTICLE III.

### *The Physical History of Various Nations of the Earth, With Special Reference to their Teeth.*

By Dr. J. Allen.

(Continued.)

The Fejeeans are generally above the middle height, and exhibit a great variety of figure. Their complexion is between that of the black and copper colored races, although instances of both extremes are to be met with, thus indicating a descent from two different stocks. The faces of the greater number of the Fejeeans are long, with large mouth, *good and well set teeth.*

Leaving the Fejee Islands, we will pass to the coast of

Australia. Captain Wilks, who was sent out by the government of the United States on an exploring expedition, says: "The natives of Australia differ from any other race of men in features, complexion, habits and language. Their color and features assimilate them to the African type, their long and black silky hair has a resemblance to the Malays; in their language they approximate more nearly to our American Indians, while there is much in their physical traits, manners and customs to which no analogy can be traced in any other people. Their color usually approaches chocolate, a deep umber, or reddish black, varying much in shade in different individuals. The cast of the face is between the African and Malay, the forehead usually high and narrow, the eyes small, black and deep set, the nose much depressed at the upper parts, the cheek bones are high, the mouth large and furnished with *strong, well set teeth.*"

We will next direct your attention to the American races or tribes of this continent. Dr. Morton who has published a very popular work on American skulls, says: "It is an old saying among travelers, that he who has seen one tribe of Indians, has seen all. So much do the individuals of this race resemble each other that notwithstanding their immense geographical distribution, and those differences of climate which embrace the extremes of heat and cold, there is a remarkable identity of physical characteristics throughout this whole race of people. All possess, alike, the long, lank, black hair, the brown or cinnamon colored skin, the heavy brow, the dull and sleepy eye, the full and compressed lips, and the salient but dilated nose."

Without following this author through his details of the physical characteristics of the American races, we will pass at once to his records of their teeth. He says: "The cheek bones are large and prominent, the upper jaw is often elongated, but the teeth are, for the most part vertical. The lower jaw is large and ponderous;

*the teeth are also very large and seldom decay*, and few present marks of disease, though often worn by the mastication of hard substances.''

With reference to the nations of the western coast of North America, we have the following record from Captain Cook and Mr. Anderson :

"The visage of most of them is rather round and full, and sometimes also broad, with high, prominent cheeks. The nose flattened at the base, the forehead is rather low, the eyes small, black and languishing rather than sparkling, the mouth round, with thickish lips, *the teeth well set* but not remarkably white.''

We will now direct attention to the nations of Chili, of California, and to those of the country near the Baie des Francais, who are of the Kolushian race. In the historical account of these people, by Mr. Rollin, we have the following: "They have rather a low forehead, black and lively eyes, nose of a regular shape and size, rather wide at the extremity ; lips fleshy, a mouth of middle size, *fine and well set teeth.*'' We will also notice the Peruvian nations. The physical characteristics of these nations in general are described by Dr. Orbigny. He says: "Their features have an entirely peculiar cast, which resemble no other American people but the Mexicans. Their head is oblong from the forehead to the occiput, somewhat compressed at the sides. The forehead is slightly arched, short, and falling a little back. Their face is generally broad, approaching to an oval form ; their nose prominent, long, and strongly aquiline ; the mouth is larger than common, though the lips are not very thick. The teeth are *always beautiful, even in old age.*'' Dr. Orbigny says the mountaineers in South America are generally short, while the inhabitants of the plains are tall. "The Aroucans are a square, stout set of men with robust limbs, but without obesity ; their joints large, their hands and feet small. Their heads are large in proportion to their body ; the countenance full, round, with prominent cheek bones, large mouths, but thin lips. *Their teeth are good, and remain sound in old age.*"

"The aboriginal nations of Eastern Patagonia," says Captain Fitzroy, "are a tall and extremely stout race of men. Their color is a rich, reddish brown. The head of the Patagonian is rather broad, but not high; the mouth is large and coarsely formed, with thick lips. Their teeth are usually *very good*, though rather large, and those in front have the peculiarity of being flattened, solid, and showing an inner substance." The following is an extract containing a description of the Pesherais, a people who inhabit one of the islands of the Magellanic Archipelago. (This extract is taken from an account of an exploring expedition sent out by the United States government.)

"These people are not more than five feet high, of a light copper color. They have short faces, narrow foreheads, and high cheek bones. Their eyes are small and unusually black. Their nose is broad and flat, with wide-spread nostrils, mouth large, *teeth white, large and regular.*" Dr. Orbigny in describing another tribe of the South American Indians, (called the Botocudos) says: "They wear for ornaments, collars or strings of human teeth." This is an evidence of their soundness and beauty. In the northern division of South America we have the following physical description of a people called the Chaymas. Humboldt has given the following description of them:

"The countenances of the Chaymas, without being hard and stern, has something sedate and gloomy. The forehead is small but slightly prominent. The eyes black, sunken, and very long. The wide mouth, with lips but little prominent, has often an expression of good nature. The nose and nostrils resemble those of the Caucasian race." "The Chaymas," says Humboldt, "have fine, white teeth, like all people who lead a very simple life."

Having taken a cursory view of the characteristics of several other nations of the world, with special reference to their teeth, we will now return to our own country. We have here a mixed population from various parts of the world, who have become so assimilated in habits, manners, customs, mode of living, etc., that the historian would recognize the same general physical characteristics of the people throughout the United States. But how different would be his record in reference to the teeth of the Americans at the present time from those nations herein referred to. He would tell you that very many of the people of this country have narrow, contracted jaws, with crowded and badly decayed teeth. And in his statistics he would announce to you the startling fact that twenty millions of teeth are annually lost by the people of this country. From the evidences which we have endeavored to bring before you, it will be seen that the teeth of the people of this country are far worse than of any other here described. Mark the words of Humboldt when he said: "The Chaymas have fine teeth, like all people who lead a very simple life." It will be observed that in these historical researches, there is no evidence that the nations Humboldt alluded to attempted to improve their food by changing the proportions of the different constituents which the Creator has duly apportioned for the building up of organized beings. But, on the contrary, those nations use their food in the most simple forms, with all the constituents which nature placed there for the use of man. Another important fact in the history of those nations who have well developed jaws and teeth, should be also noted. It is this: they have plenty of exercise in the open air, which enables them to appropriate the different constituents in their food to the various parts and organs of the human system. From these different nations, therefore, we may learn some valuable lessons on the subject of the teeth. Although they have no Dentists nor Dental literature, (for they need none) yet they learn much, as we may, from Nature, which will be found to tally exactly with true science.

Now let us turn again to our own records and see how widely we have departed from some of those physical laws which have been established by Omnipotence for our well-being. We have vainly attempted to improve our bread (the staff of life) by changing the proportions of the mineral element in the flour we use, by bolting the most of it out and discarding it. Look for a moment at the gigantic scale upon which it is done in this country. According to our national statistics of 1860, there were in the United States, 13,868 milling establishments for the manufacture of flour and meal, requiring 27,626 men, at an annual cost for labor of 8,721,391 dollars. Thus, you see, the number of men, mills, bolting cloths and dollars that are employed in this great *improvement* (?) devised by man for changing the proportions of one of the most important constituents in the staple article of food in this country. The result of ignoring this mineral element from the staff of life is, undoubtedly, to a great extent one of the most prominent causes of this national calamity, that sweeps from the population more than 20,000,000 of teeth every year. The potter cannot make the bowl without the clay, neither can good teeth be formed without a due proportion of lime, which is abundantly provided for our use upon the outer portion of the grain, and in rejecting this portion of the cereals we virtually refuse to use the requisite materials of which the teeth are formed. We also deprive ourselves of a due proportion of atmospheric constituents, especially in our crowded cities. And also of the requisite amount of exercise to promote vigorous health and good constitutions. If we would be instrumental in doing more good in our profession, let us do all in our power to diffuse these important truths among the people.

